# Towards a mechanistic understanding of particle shrinkage during biomass pyrolysis via synchrotron X-ray microtomography and in-situ radiography

**DOI:** 10.1038/s41598-020-80228-x

**Published:** 2021-01-29

**Authors:** Meredith Rose Barr, Rhodri Jervis, Yeshui Zhang, Andrew J. Bodey, Christoph Rau, Paul R. Shearing, Dan J. L. Brett, Maria‐Magdalena Titirici, Roberto Volpe

**Affiliations:** 1 Division of Chemical Engineering and Renewable Energy, School of Engineering and Materials Science, Queen Mary University of London, Mile End Road, London, E1 4NS UK; 2 Electrochemical Innovation Lab, Department of Chemical Engineering, Faculty of Engineering Sciences, University College London, Gower Street, London, WC1E 7JE UK; 3 Diamond Light Source, Harwell Science & Innovation Campus, Didcot, OX11 0DE UK; 4 Department of Chemical Engineering, Faculty of Engineering, Imperial College London, South Kensington Campus, London, SW7 2AZ UK

**Keywords:** Chemical engineering, Porous materials

## Abstract

Accurate modelling of particle shrinkage during biomass pyrolysis is key to the production of biochars with specific morphologies. Such biochars represent sustainable solutions to a variety of adsorption-dependent environmental remediation challenges. Modelling of particle shrinkage during biomass pyrolysis has heretofore been based solely on theory and ex-situ experimental data. Here we present the first in-situ phase-contrast X-ray imaging study of biomass pyrolysis. A novel reactor was developed to enable *operando* synchrotron radiography of fixed beds of pyrolysing biomass. Almond shell particles experienced more bulk shrinkage and less change in porosity than did walnut shell particles during pyrolysis, despite their similar composition. Alkaline pretreatment was found to reduce this difference in feedstock behaviour. Ex-situ synchrotron X-ray microtomography was performed to study the effects of pyrolysis on pore morphology. Pyrolysis led to a redistribution of pores away from particle surfaces, meaning newly formed surface area may be less accessible to adsorbates.

Lignocellulosic biomass (LCB) is currently the largest stream of non-edible biomass globally, the primary sources of which are agricultural and forestry waste streams^1^. LCB is naturally porous, and thus has a high surface area to volume ratio. This allows it to adsorb a large quantity of small molecules, materials, or organisms relative to less porous materials of similar bulk volume. However, upon heating LCB without addition of an oxidising atmosphere, it undergoes a complex transformation that is known to vastly increase its adsorptive surface area^2–5^. The process leading to this transformation is known as pyrolysis, and the result as charcoal, char, or biochar.

Biochar is known as a universal adsorbent, meaning it can adsorb a wide variety of materials. It is used for a range of environmental remediation applications such as air, water, and soil treatment, as well as carbon capture and storage^6^. Particularly interesting is its potential for retention of organisms and molecules that contribute to environmental antimicrobial resistance (AMR). AMR is the phenomenon whereby microorganisms begin to tolerate compounds which would otherwise kill them or prevent their growth (antimicrobials)^7,8^. According to a report commissioned by the UK government, infections resistant to clinical antimicrobials caused approximately 700,000 deaths per year as of 2016, and are predicted to cause 10 million deaths per year by 2050^9^. While this is largely driven by clinical (over/mis)use of antimicrobials, it is also driven by the interaction of microorganisms with antimicrobials (via selective pressure) and resistant microorganisms (via horizontal gene transfer)^10^ in the environment^11,12^. Biochars have been shown to be effective at removing bacteria^13–18^ and antimicrobials^19–28^ from soil runoff and wastewater.

The efficacy of biochars for these adsorption applications relies heavily on char morphology. Beyond simply maximising adsorbent surface area, adsorbates must be able to access the internal surface area of particles, which is dependent on pore and particle morphology. Pore morphology is influenced by a number of factors, including diameter, tortuosity, and connectivity. Particle morphology is affected by the distribution of these pores within the particle, as well as bulk morphology (size and shape). During pyrolysis, both particle and pore morphology evolve. These processes are commonly referred to as particle shrinkage, though in reality this term encompasses two parallel processes: bulk particle shrinkage and porosity gain.

“What causes particle shrinkage and how can it be predicted?” is considered one of the top ten fundamental challenges of biomass pyrolysis for biofuels^29^. Properly accounting for bulk particle shrinkage and porosity gain based on direct observation of morphological evolution could lead to unprecedented accuracy and detail of biomass pyrolysis models. Such advanced models would allow char morphology to be tailored for specific adsorption applications. This is particularly useful for adsorption of microorganisms, given the enormous variety in morphology, surface chemistry, and motility of such organisms.

There are two conventional varieties of model for particle shrinkage: uniform conversion (UC) models, also called volumetric decomposition models; and shrinking unreacted particle (SUP) models, also called shrinking core models. UC models assume that porosity is infinitely fine and evenly distributed throughout particles, and therefore that the entire volume of the particle is in good contact with the atmosphere. Under this assumption, the particle’s external surface area is negligible compared to its internal surface area. Therefore, this model predicts volume loss to be purely internal; that is, all volume lost by the solid results in porosity gain. Conversely, SUP models assume that only a thin external layer of the particle is in good contact with the atmosphere. As a result, volume loss is purely external; that is, all volume lost by the solid results in bulk particle shrinkage^30,31^.

Contrary to both conventional models, it is known that internal and external volume loss occur simultaneously during biomass pyrolysis. To account for this, there have been several attempts to combine the approaches of these conventional models^30–36^, but validation of these attempts is lacking due to the challenging nature of imaging under controlled pyrolysis conditions. Several studies have employed post-pyrolysis imaging (both external^37,38^ and internal^2,4,39^) to study the evolution of particle morphology during pyrolysis, but these neglect the effects of cooling and recovering chars for analysis. It is recognised that “In situ imaging… will provide unprecedented and much-needed information for developing a new generation of models”^29^. While several researchers have managed to externally image pyrolysing biomass particles in situ using digital cameras^40–43^, no internal imaging studies have been performed in situ prior to this study.

This work attempts to bridge this gap, using a combination of in-situ and ex-situ synchrotron X-ray imaging techniques, in phase-contrast mode, to study the evolution of particle and bed morphology during biomass pyrolysis. X-ray imaging is a non-destructive characterisation technique that, when using high-brilliance synchrotron sources delivering spatially coherent X-ray beams can yield high temporal, spatial, and contrast resolution of evolving microstructures. This has recently been used to great effect to study electrochemical devices and how their microstructures change during operation or failure^44^. As in the present work, both 2D radiographs and 3D tomograms (constructed from a series of radiographs taken at angular increments of sample rotation) were used. Fewer examples of the application of X-ray radiography or tomography to biomass exist in the literature, though some examples of ex-situ microtomographic imaging of biomass are available^2,4,39^.

In order to study the effects of feedstock morphology and composition on particle shrinkage, two types of nut shells, almond and walnut, were considered as pyrolysis feedstocks. These nut shells are both agricultural waste products, and are known to have relatively similar chemical compositions and microscale morphologies, but to differ in macroscale morphology in that almond shells contain macroscopic vascular channels, while walnut shells do not^45^. In addition to pyrolysis of raw biomass, that of biomass treated with NaOH (known to increase porosity and reduce lignin content)^46–49^, as well as that of biomass soaked in water (known to wash away external inorganic compounds)^30,50^ have been studied. For the first time, a pyrolysis reactor allowing the evolution of particle morphology to be observed in situ by synchrotron X-ray imaging has been developed, giving unprecedented insight into the behaviour of biomass during pyrolysis.

## Results

### In-situ radiography

**Figure 1:**
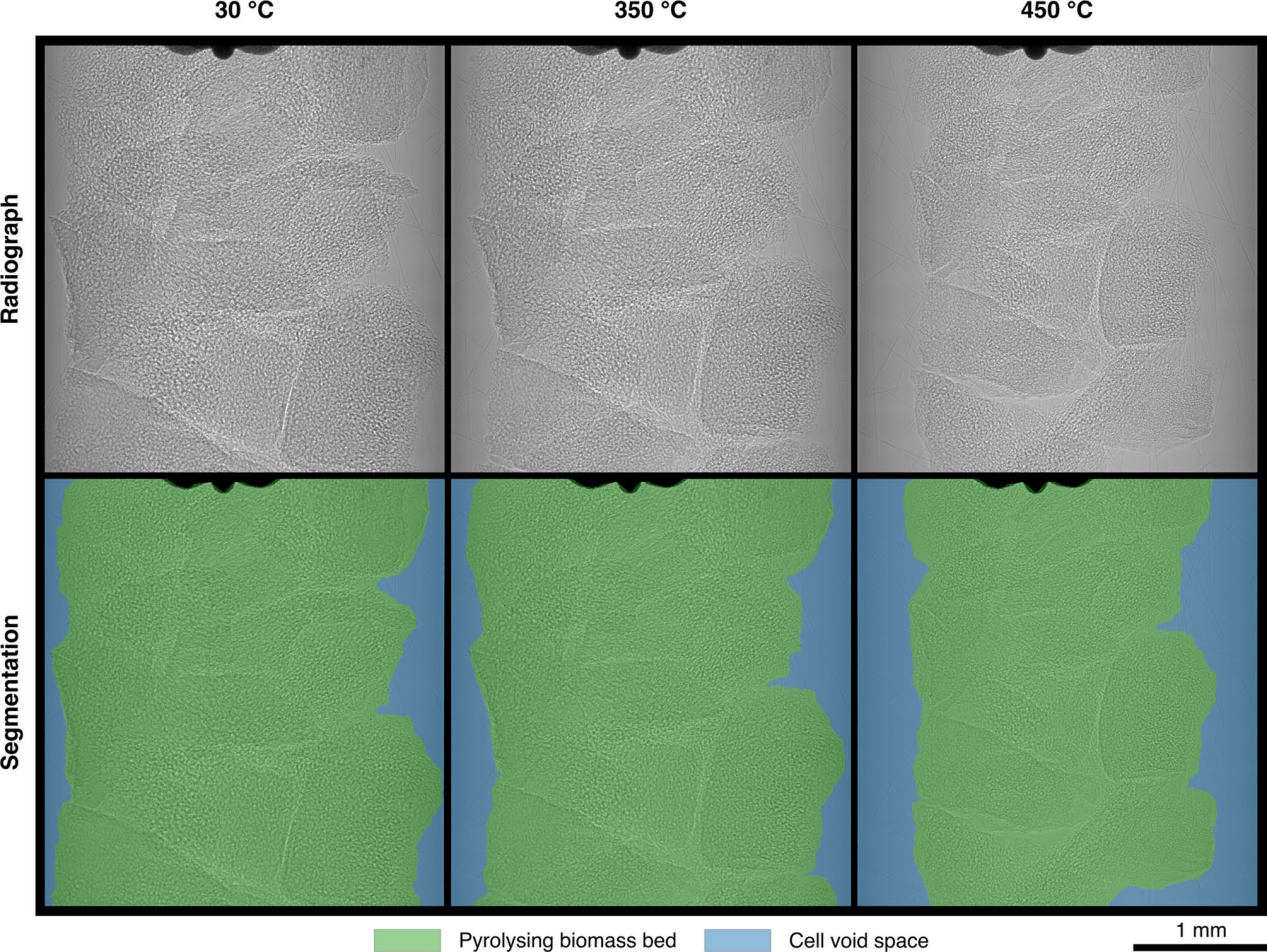
Radiographs and segmentations of a bed of water-soaked almond shells undergoing pyrolysis at a heating rate of 6 °C min^−1^. Radiographs were acquired using a pink synchrotron X-ray beam (weighted mean energy ~ 27 keV) with effective pixel size of the detector set as 1.625 μm with an exposure time of 0.05 s.

Examples of acquired in-situ radiographs, and segmentations thereof, can be seen in Fig. 1. Percentage changes and rates of change of two parameters were considered in the analysis of these:

1. Fraction of the total available cell cross-sectional area (CSA) occupied by the pyrolysing bed.
2. Average pixel value (APV) in terms of 8-bit intensity with respect to that of the void cell CSA (available cell area not occupied by the pyrolysing bed). Greater APV indicates a lighter image, and therefore less X-ray attenuation of the bed and/or more phase contrast.

Metric (1) may be interpreted as a measure of bulk bed shrinkage, which includes both individual bulk particle shrinkage and increased particle packing density as pyrolysis proceeds. Metric (2) encompasses several phenomena. Pixel intensity is determined both by the degree of X-ray attenuation, which relates to chemical composition and material density; and by X-ray phase-contrast, which highlights boundaries between different phases, in this case solid–gas interfaces^51^. Here, APV is primarily affected by changes in morphology, since the dramatic particle shrinkage observed (see Fig. 1) results in increased material density of the bed, and porosity gain in more solid–gas interfaces. As particles shrink in the stream of gas, they increasingly overlap, increasing the bed packing density (and therefore material density), thus lowering the bed APV. However, as particles gain porosity, their APV increases as bulk particle density decreases and internal surface area increases. Therefore, the change in metric (2) as pyrolysis proceeds is a good indication of whether bulk particle shrinkage (indicated by a decreasing trend) or porosity gain (indicated by an increasing trend) dominates. This can be equivalently described as whether particles behave more similarly to SUP or UC models of particle shrinkage, respectively.

**Figure 2:**
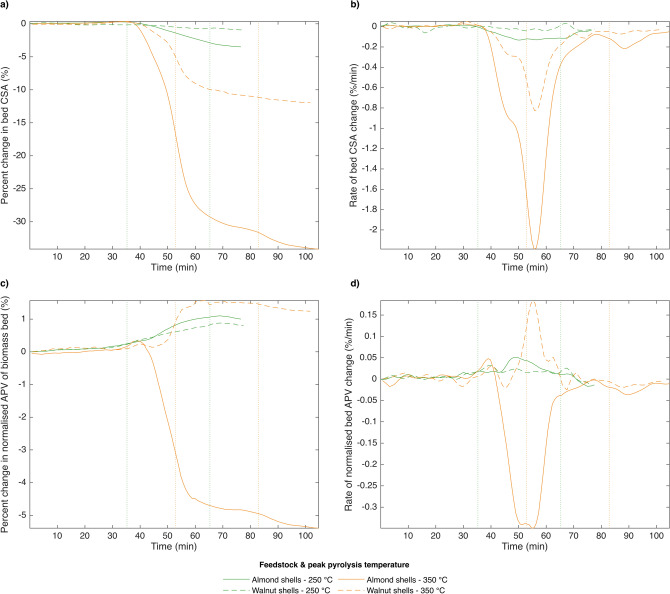
Evolution of cross-sectional area (CSA) and normalized average pixel value (APV) of untreated biomass beds by feedstock and peak pyrolysis temperature. APV of the biomass bed is temporally normalised by that of the cell void space. Samples were pyrolysed by a 3 L min^−1^ stream of pre-heated argon at a rate of 6 °C min^−1^, followed by a 30-min hold time at peak temperature. Vertical lines indicate the start and end of the 30-min hold time. (**a**) CSA. (**b**) d(CSA)/dt. (**c**) APV. (**d**) d(APV)/dt.

**Figure 3:**
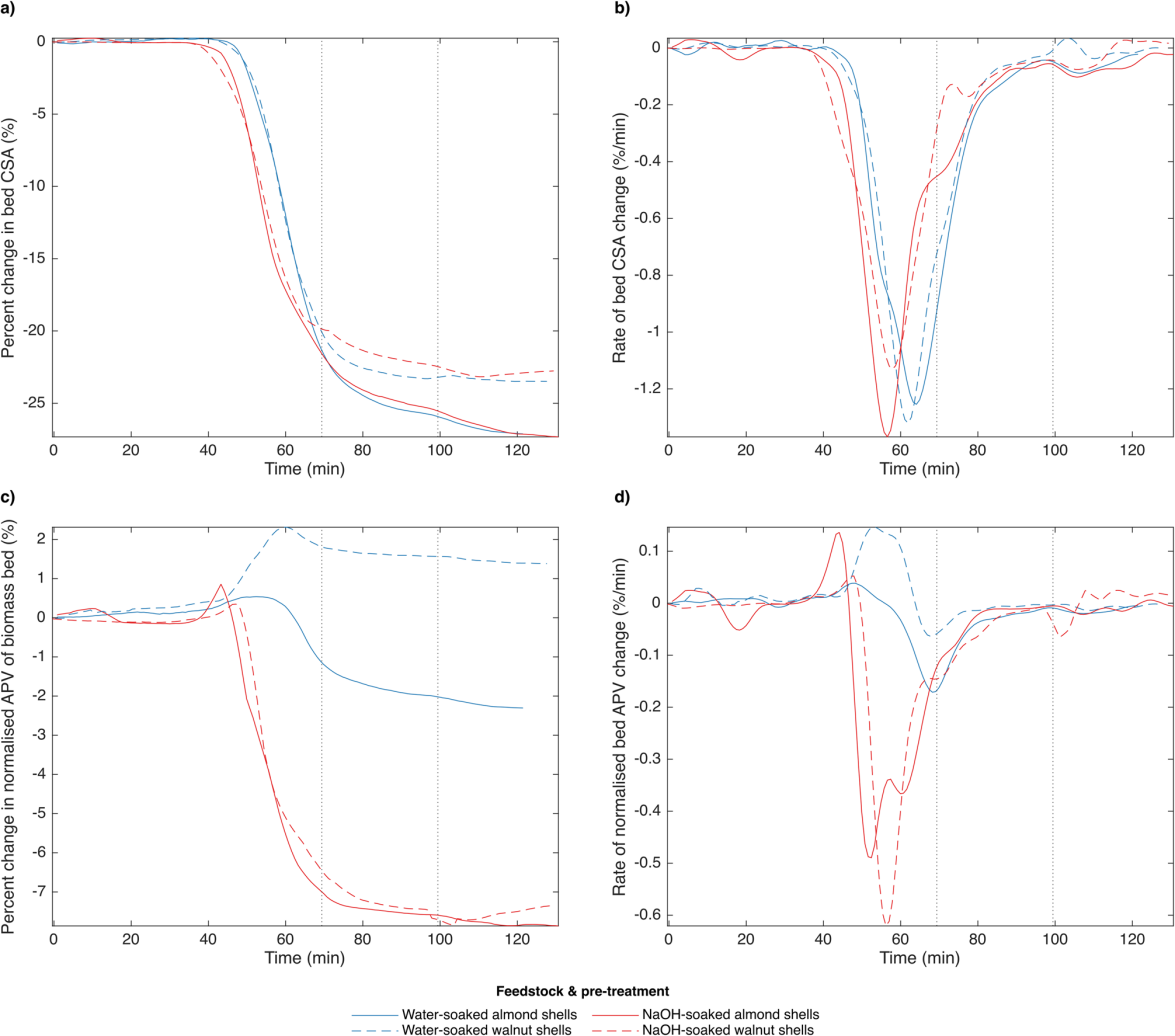
Evolution of cross-sectional area (CSA) and normalized average pixel value (APV) of pretreated biomass beds by feedstock and method of pretreatment. APV of the biomass bed is temporally normalised by that of the cell void space. Samples were pyrolysed by a 3 L min^−1^ stream of pre-heated argon at a rate of 6 °C min^−1^, followed by a 30-min hold time at peak temperature. Vertical lines indicate the start and end of the 30-min hold time (450 °C for all samples). (**a**) CSA. (**b**) d(CSA)/dt. (**c**) APV. (**d**) d(APV)/dt.

Figures 2 and 3 show results of radiograph analyses for untreated and treated samples, respectively. In each figure, trends in metric (1)—bed CSA—over the course of the pyrolysis reaction are shown in the top row, and those in metric (2)—bed APV—in the bottom row. The measured metrics over time are presented in the left column, and their derivatives in the right column, in order to highlight regimes and maximum rates of morphology change. These figures reveal two key findings. Firstly, almond shell particles were consistently found to shrink more than were walnut shell particles, irrespective of pretreatment, while walnut shell particles were found to favour porosity gain more than were almond shell particles. Secondly, pretreatment with NaOH led both feedstocks to shrink at a lower temperature than untreated biomass, and to favour bulk shrinkage to porosity gain.

In greater detail, bed CSA of untreated almond shells was shown to decrease substantially more than that of untreated walnut shells when pyrolysed at peak temperatures of 250 and 350 °C (see Fig. 2a). The same was shown to be true of treated nut shells pyrolysed at a peak temperature of 450 °C (see Fig. 3a). Untreated shells pyrolysed at a peak temperature of 450 °C are not included here simply because they were scanned only at a higher magnification, which prevented analysis of the full biomass bed. Shells which were pretreated with NaOH prior to pyrolysis shrank at a lower temperature than did those soaked only in water for both feedstocks considered. However, pretreatment method did not affect the overall magnitude of bed shrinkage, which was instead determined by feedstock, as in untreated shells (see Fig. 3a).

In considering the derivative plots (Figs. 2b and 3b), note that greater peak intensity indicates a faster rate of change, while greater peak width indicates more sustained change. Figure 2b shows that the rate of bed CSA change of untreated nut shells consists of two distinct regimes for both feedstocks, changing from a slower to a faster rate around 320–325 °C. The maximum rate of bed shrinkage of these samples occurred during the 30-min hold at peak temperature (350 °C). This same rate-duality was not consistently observed in treated shells (see Fig. 3b), for which the rate of bed shrinkage was found to peak between 373 and 381 °C for NaOH-soaked samples and between 404 and 418 °C for water-soaked samples. This difference in peak morphology could be due to the absence of lignin in the NaOH-soaked samples, or to their greater initial porosity, but in this case, two regimes would still be expected from water-soaked samples, which are not clearly observed. This suggests the effect could be related to the role of inorganics in biomass pyrolysis, since both pretreatments involve washing away external inorganic compounds with water. The onset of bed shrinkage was observed to occur between approximately 250 and 300 °C for all samples.

During pyrolysis to a peak temperature of 350 °C, normalised APV was shown to increase for walnut shells, but to decrease for almond shells (see Fig. 2c). The same was shown to be true of water-soaked shells pyrolysed at a peak temperature of 450 °C, but not of NaOH-soaked shells, for which normalised APV decreases similarly for both feedstocks (see Fig. 3c). Despite their opposite trends, the peak rates of normalised APV change for untreated and water-soaked almond and walnut shells appear to occur at similar temperatures, similar to their maximum rates of bed shrinkage (see Figs. 2d and 3d). Comparing Figs. 2b and 3b to Figs. 2d and 3d shows that the range of temperatures over which the majority of change in the two metrics considered occurs is similar among all samples. The samples that increase in normalised APV while decreasing in bed CSA are a good indication that bulk particle shrinkage and porosity gain occur concurrently during pyrolysis, a fact that is not captured by either conventional method of modelling particle shrinkage.

All samples show a local maximum in normalised APV towards the beginning of their period of change in this metric (see Figs. 2c and 3c). This could indicate either the occurrence of swelling and subsequent shrinking of pores, or that there is a point at which bed packing densification begins to outweigh porosity gain as pyrolysis proceeds. The majority of samples also show a slight increase in bed CSA before bulk shrinkage occurs, but the magnitude of this increase is not great enough to conclusively say it is due to swelling rather than segmentation noise or subtle particle movement. Despite this, several existing studies of particle shrinkage have observed evidence of swelling during biomass pyrolysis of relatively large particles^37,38,41,43^.

Overall, these results indicate that both the magnitude and mechanisms of particle shrinkage vary even among relatively similar feedstocks, and that their difference in the evolution of porosity during pyrolysis may be reduced by alkaline pretreatment. Though results confirmed that bulk particle shrinkage and porosity gain occur simultaneously during biomass pyrolysis, solid volume loss in almond shells more closely resembled that of an SUP model, wherein all solid volume loss results in bulk particle shrinkage, while that in walnut shells more closely resembled that of a UC model, wherein all solid volume loss results in porosity gain. One explanation for this, further explored in the discussion, could be a difference in the rate of heat transfer into feedstock particles caused by their different morphologies.

### Tomography

**Figure 4:**
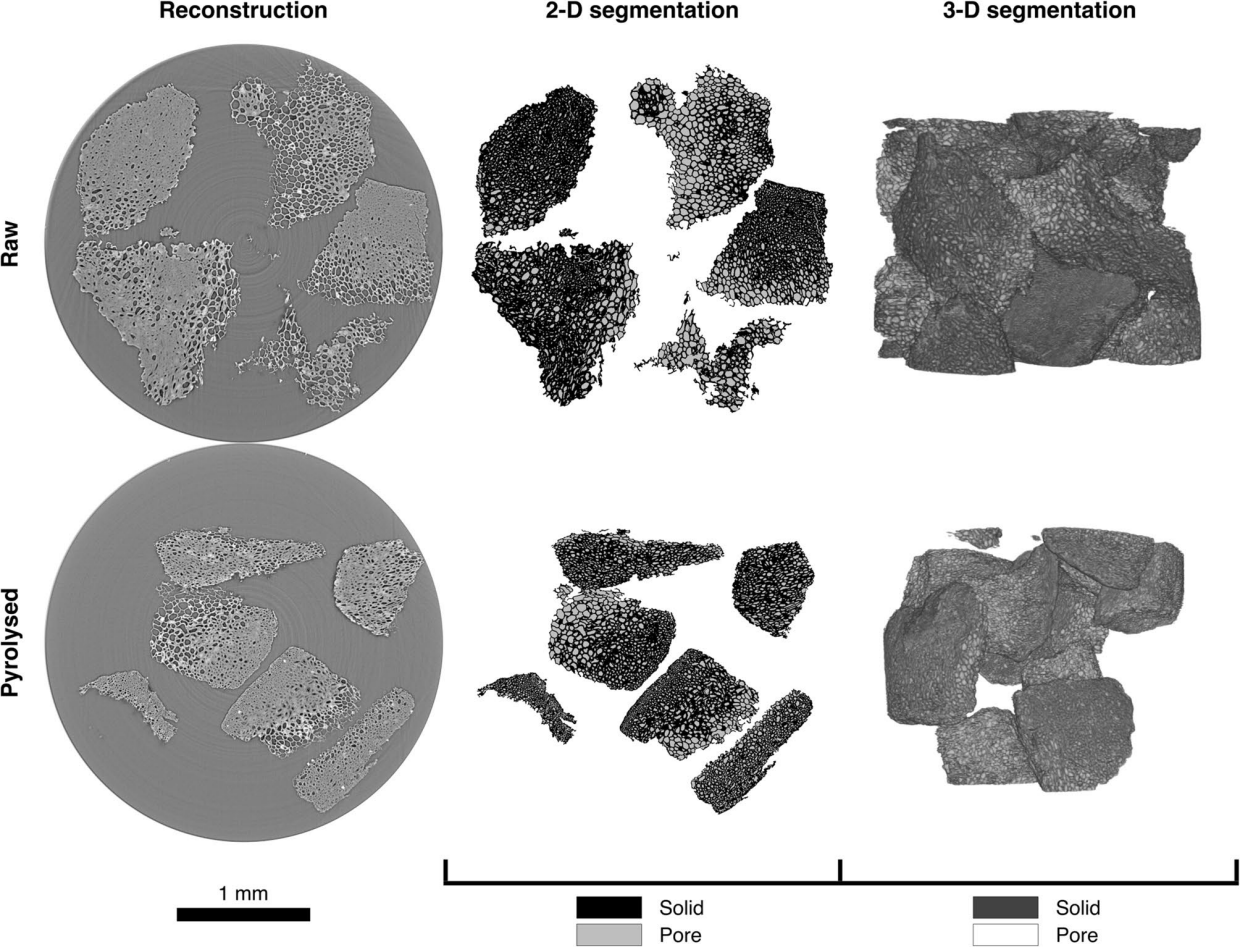
Reconstructed and segmented tomograms and corresponding three-dimensional (3-D) renderings of raw and pyrolysed water-soaked almond shells. Pyrolysed shells were heated at a rate of 6 °C min^−1^ to a peak temperature of 450 °C and held for 30 min. In 3-D renderings, the scale bar corresponds to the frontmost plane.

**Figure 5:**
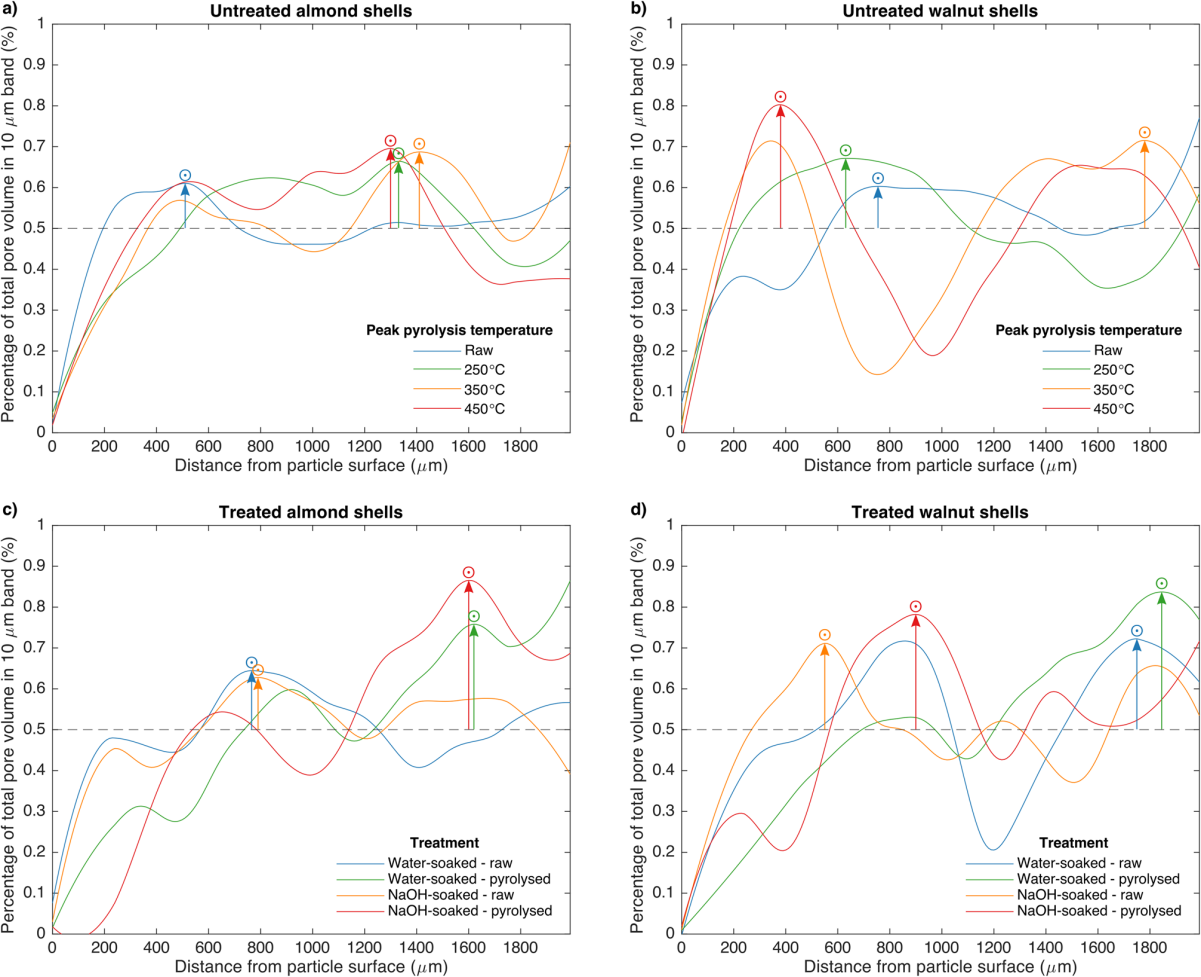
Distributions of pores in biomass beds with respect to the distance of their centres of mass from the nearest exposed particle surface for (**a**) untreated almond shells, (**b**) untreated walnut shells, (**c**) pretreated almond shells and (**d**) pretreated walnut shells. The volume of pores in each of two hundred 10 μm wide bins were summed to give an indication of where within particles the bulk of pore volume is located with respect to exposed surfaces. The greatest local maximum of each distribution is indicated by the symbol ⨀. The displacements of these maxima from the “equal distribution line” at 0.5%, normalized by the greatest observed value of this displacement, are termed “degree of pore concentration”, and represented by the symbol ⨀, the values of which are plotted in Fig. 7.

Examples of reconstructed tomograms, and segmentations thereof, can be seen in Fig. 4. Minimum distances between each individual pore in three-dimensional segmentations and the nearest particle surface were calculated. Voxels identified as pore space were grouped into individual pores using a 6-way pixel connectivity rule—that is, only pixels touching at a surface were considered connected. Pore locations were determined by their centroids, or centres of mass assuming constant density. Large continuous networks of pores—those more than 1 standard deviation greater than the mean pore volume—were excluded because their centroids would not accurately describe their location. The total pore volume in each 10 μm band of distance from a particle surface was then plotted with respect to this distance. The resulting distributions (Fig. 5) describe whether pore volume is generally concentrated closer to or further from particle surfaces in the sample bed. This is an important characteristic in determining the ability of adsorbates to access internal surface area, and therefore in determining biochar adsorption capacity.

When pores are more concentrated with respect to their distance from particle surfaces, the distribution shows distinct peaks. Conversely, when pores are evenly distributed throughout the particle, the distribution should decrease with distance from the particle surface, since the total particle volume contained in the 10 μm band decreases with this distance. However, all samples show a minimum at the particle surface. Two effects are at play here, which can be seen in the 2-D segmentations in Fig. 4. Firstly, in dense regions of particle, bright bands appear near particle surfaces. This is an effect of the phase-contrast-enhanced imaging, which could be obscuring surface pores. Alternatively, there may actually be fewer surface pores in dense regions because heat is less able to penetrate particles there. This would lead to a distinct surface region in which kinetics are faster than heat transfer, and the particle reacts away mainly at the surface, shrinking back rather than gaining porosity. Deeper into such regions, the lower temperature would result in heat transfer outpacing kinetics, meaning the particle would react throughout its volume, leading to porosity gain greater than that at the surface. Secondly, in less dense regions, pores are larger. Even though they are close to particle surfaces, they are recorded as further because their centroids are further than would be those of smaller pores. Unfortunately, calculating distances from the nearest pore surface to the nearest particle surface would be orders of magnitude more computationally demanding.

**Figure 6:**
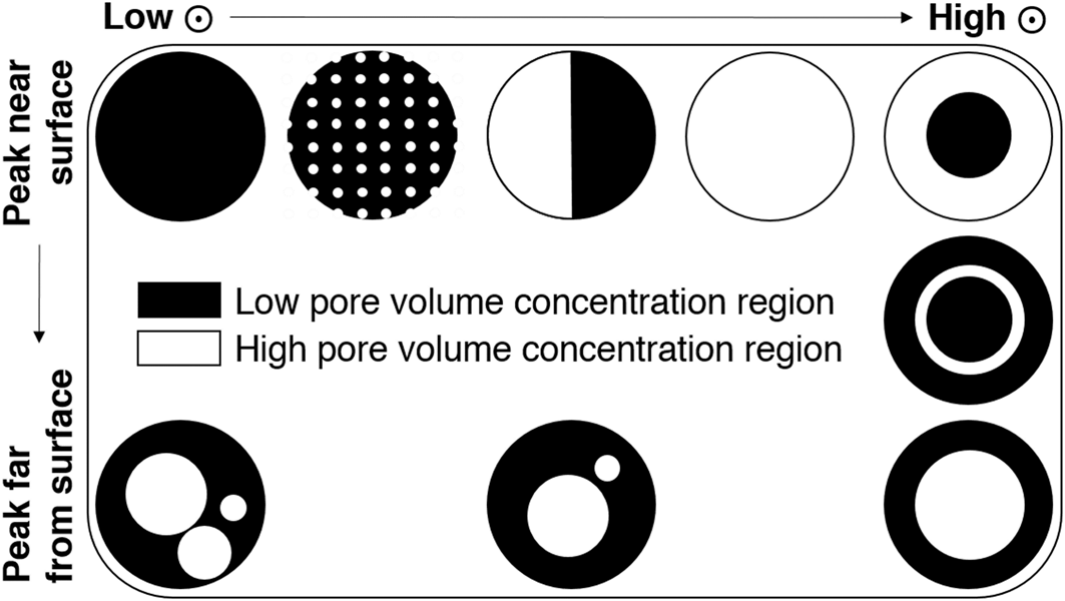
Visual representation of the meanings of peaks in pore volume with respect to distance from the nearest particle surface. ⨀ is related to peak height. Simplified example distributions are presented here to aid in interpretation of Figs. 5 and 7.

Distributions were plotted up to 2 mm from the particle surface, which is the maximum particle size, since this is the maximum distance a pore could reasonably be from a surface, even when accounting for cropping errors (cropped edges are not considered particle surfaces in calculations). Because two hundred 10 μm bands were considered, a flat distribution at 0.5% represents an equal volume of pores in each band. Notably, this equal distribution of pore volume is not equivalent to an even distribution of pore volume throughout the particle, which would lead to a peak in pore volume at the particle surface, as discussed above. Therefore, while a peak at the surface can signify either an even distribution of pore volume or a concentration of pore volume towards particle surfaces, a peak elsewhere clearly signifies a concentration of pore volume in this region. Figure 6 provides examples to guide interpretation of these distributions. Begin by considering the “ideal” situations described in the rightmost column, then consider the situations presented moving leftwards as examples of how surface peak meaning may be convoluted, while peaks far from particle surfaces retain their meaning regardless of intensity.

**Figure 7:**
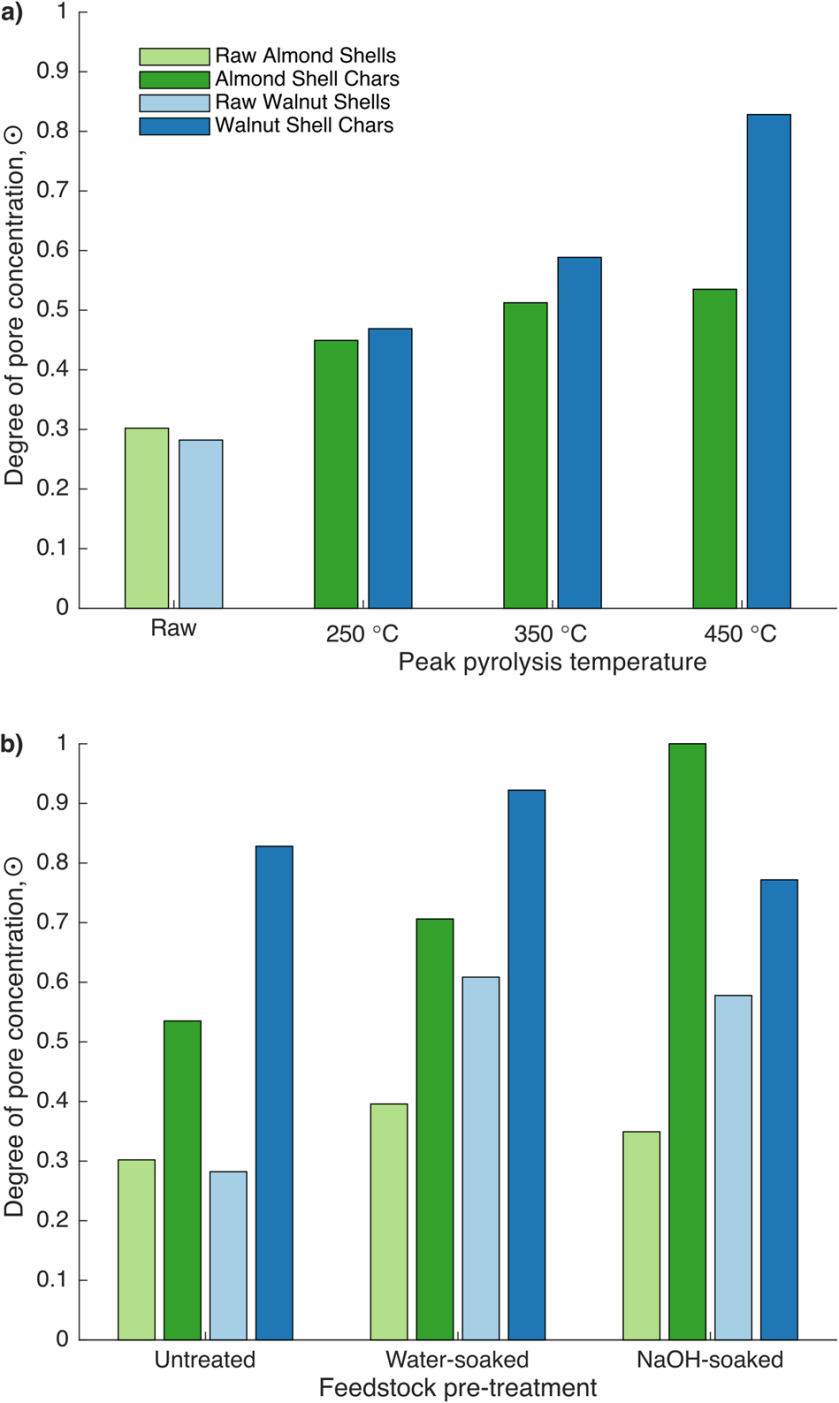
Degree of pore concentration, ⨀, of (**a**) untreated raw biomass and biochar beds by peak pyrolysis temperature and feedstock and (**b**) raw biomass and biochar beds produced at a peak pyrolysis temperature of 450 °C by pretreatment method and feedstock.

Because tomography was performed ex-situ, and biomass begins with an effectively random distribution of pores, the distributions presented in Fig. 5 cannot be directly compared to one another. Despite this, the height of the maximum peak in each distribution, which represents a measure of the degree to which pores are concentrated with respect to distance from a particle surface, trends upward with peak pyrolysis temperature, and is consistently greater in pyrolysed samples than raw samples for both treated and untreated shells (see Fig. 7). This metric, calculated as the displacement of the maximum peak in pore volume with respect to distance from a particle surface from the equal distribution line (0.5%), normalised by the greatest observed value of this displacement, will be termed the degree of pore concentration, and represented by the symbol ⨀.

While Fig. 7 shows that the degree of pore concentration, ⨀, increases upon pyrolysis of nut shells, Fig. 5 reveals more about the way in which pores concentrate in different feedstocks. In almond shells, ⨀ consistently occurs substantially further from particle surfaces in pyrolysed than raw samples. The shift is similar among all treatments and temperatures. Though the same trend holds for treated walnut shells, the shift is both less extreme and less consistent between conditions. Conversely, in untreated walnut shells, peaks appear both near particle surfaces and centres in pyrolysed samples. This could either imply (1) redistribution of pores towards particle surfaces and centres, or (2) redistribution towards centres coupled with a substantial general increase in porosity throughout particles (due to the greater surface area of surface bands). Because in-situ radiographs showed that walnut shells increase in porosity more than do almond shells during pyrolysis, it is likely that explanation (2) is correct, and all feedstocks and conditions simply concentrate pore volume towards particle centres during pyrolysis. This would subsequently imply that the extent to which porosity increases during pyrolysis varies among these similar feedstocks, and is affected by pretreatment, which is consistent with conclusions drawn from in-situ radiographs.

Furthermore, untreated walnut shells appear to increase in ⨀ substantially more than untreated almond shells between raw and pyrolysed samples, implying they experience more extreme redistribution of pore volume during pyrolysis. This is consistent with the assessment of the radiography results that walnut shells behave more similarly to UC models of particle shrinkage, wherein only internal volume changes, while almond shells behave more similarly to SUP models, wherein volume is lost only at particle surfaces. However, since all feedstocks and conditions showed greater ⨀ in pyrolysed than raw samples, implying some internal volume redistribution during pyrolysis, while radiographs clearly showed bulk particle shrinkage, tomograms further support the conclusion that internal and external solid volume loss occur simultaneously during pyrolysis. Furthermore, tomograms support the conclusion that the proper mix of these models is dependent on the feedstock, even among biologically and chemically similar feedstocks.

## Discussion

The following conclusions may be drawn with respect to slow pyrolysis of almond and walnut shells, bearing in mind that the results may be specific to the sample, experimental, and reactor conditions described in the Methods section^52^:

1. External and internal solid volume loss (bulk particle shrinkage and porosity gain) occur simultaneously during pyrolysis.

This fact was first captured by Di Blasi^32^ in their 1996 model of pyrolysis in a shrinking biomass particle using a mixing parameter that dictates the portion of solid volume loss which manifests as porosity gain as opposed to bulk shrinkage. Many models which account for this behaviour since have been based on this model^30,33–36^. More recently, Gentile et al.^31^ have accounted for this mix changing as both the rates of heat transfer and chemical kinetics change during pyrolysis by modelling a particle with an outer region following an SUP model, and an inner region following a UC model. The front between these regions then progresses as heat penetrates the particle.

(2) Pyrolysis leads to a redistribution of pore volume away from particle surfaces.

In sufficiently large particles, heat-transfer limitations dictate that the centre of the particles be colder than particle surfaces during pyrolysis. At low temperatures, and therefore towards particle centres, heat transfer is faster than chemical kinetics, and thus particles behave similarly to a UC model, primarily gaining porosity as mass is lost. At higher temperatures, and therefore towards particle surfaces, chemical kinetics are faster than heat transfer, and thus particles behave similarly to an SUP model, losing volume primarily at the surface. Therefore, porosity must accumulate at the centre of particles during pyrolysis. This phenomenon was predicted by the particle shrinkage model of Gentile et al.^31^, but has never been demonstrated experimentally prior to this study.

(3) Almond shell particles experience more change in bulk volume and less change in pore volume than do walnut shell particles during pyrolysis, despite their similar composition.

One explanation for this difference could be that walnut shells experience greater heat-transfer limitations than do almond shells. This would lead to a more persistent central particle region in which solid volume loss primarily manifests as porosity gain. This may be explained by a macroscopic difference in the morphology of the two nut shells. While they are very similar in terms of chemical composition and microscale morphology, the structure of the shells, and thus the morphology of particles, differs between the two species. Both walnut and almond shells consist primarily of thick-walled stone cells, with regions of thinner-walled cells near surfaces. The primary morphological difference is that almond shells contain macroscopic vascular bundle channels, while walnut shells are relatively solid on a macroscopic scale (see Queirós et al.^45^ Fig. 1). Thus, almond shells have far more surfaces and therefore far more thin-walled cells, making them more conducive to internal convection. This was confirmed by tomography of the raw feedstocks, which showed that raw almond shells were approximately twice as porous (28% v/v) as raw walnut shells (14% v/v).

(4) Alkaline pretreatment appears to reduce effect (3).

Since the composition of lignins in both feedstocks is similar^45^, this is likely the result of the increase in porosity caused by this treatment. If porosity of both samples essentially reached saturation, then the morphological distinction described above would no longer apply, and both feedstocks would be expected to mainly shrink rather than gain porosity during pyrolysis. Figure 3a,c show that NaOH-soaked samples shrink at a lower temperature and more strongly favour bulk shrinkage to porosity gain than do water-soaked samples. This supports the explanation that the effect of the pretreatment with NaOH is due to its impact on initial porosity of the feedstocks, as well as that for effect (3)—that almond shells are naturally more porous than walnut shells—because the pretreatment changes the behaviour of walnut shells more than it does almond shells. This is further supported by Fig. 7b, which shows that chemical pretreatments affect the degree of pore concentration in raw walnut shells more than they do in raw almond shells.

The observed concentration of pores, and therefore adsorptive surface area, towards the centre of particles during pyrolysis poses a challenge to optimising their adsorption capacity. Feedstocks with lower initial porosity have more potential for increased surface area upon pyrolysis. However, although surface area increases as pyrolysis proceeds, so too does it become increasingly inaccessible. One solution might be pretreatment of biomass with NaOH, which increases feedstock surface area prior to pyrolysis, thus reducing the heat transfer limitations likely to be driving the concentration of pores towards particle centres. Despite this, particles pretreated with NaOH in this study were still found to concentrate pores towards particle centres during pyrolysis. Another solution could be milling particles after pyrolysis when using the produced biochars for adsorption applications.

Future work will focus on two key tasks: developing a system for in-situ X-ray imaging of biomass pyrolysis in three dimensions, and linking observed morphologies to microbial and antimicrobial adsorption characteristics. Improving the experimental setup to allow acquisition of three-dimensional in-situ results will expand the insight gained from this study by permitting direct comparison and tracking of three-dimensional morphologies. The goal of this is to propose detailed mechanisms of particle shrinkage. Linking biochar morphologies and associated process conditions to their ability to adsorb and retain microorganisms and antimicrobials will enable us to tailor biochar morphologies for essential medical and environmental applications through thoughtful choice of process conditions.

## Methods

### Materials

Almond and walnut shells sourced from Italy were chosen as feedstocks for their relative homogeneity, as well as their similar chemical composition. Similar feedstocks have been thoroughly characterised by Queirós et al.^45^ These shells represent the stony lignacious endocarp of the fruit, with lignin contents of around 30%^45^. Their brittleness makes it possible to effectively mill them to the relatively large particle sizes of interest here without excessive production of fines, unlike many primarily cellulosic biomass feedstocks.

### Sample preparation

Almond and walnut shells were milled using a 2 mm grate in a Retsch ZM 200 Ultra Centrifugal Mill. A particle size of 1–2 mm was then obtained using a 1 mm sieve at an amplitude of 1.7 mm for 6 min in a Retsch AS 200 Vibratory Sieve Shaker. Shells were then dried for 48 h at 105 °C. Some shells then underwent further pre-pyrolysis treatment; treated shells were soaked at a concentration of 50 g L^−1^ in either deionized (DI) water or 200 mM NaOH for 68 h at room temperature, then washed with DI water until neutral filtrate pH was achieved. Water-soaked samples were washed the same number of times as their base-soaked counterparts for consistency. Treated shells were then dried for 48 h at 105 °C. This method is based on those used by Misson et al.^46^ and Sharma et al.^47^ to reduce the lignin content of biomass for decomposition processes by solubilizing and washing away the lignin. This process is also known to increase biomass porosity^46–49^. Here, the intention is to study the effects of lignin content and initial porosity on the evolution of morphology during pyrolysis without the added convolution of using a different feedstock. Water-soaked samples were included as a control for the effects of soaking the biomass, which is known to remove some external impurities, including inorganics^30,50^.

### Pyrolysis and image acquisition

**Figure 8:**
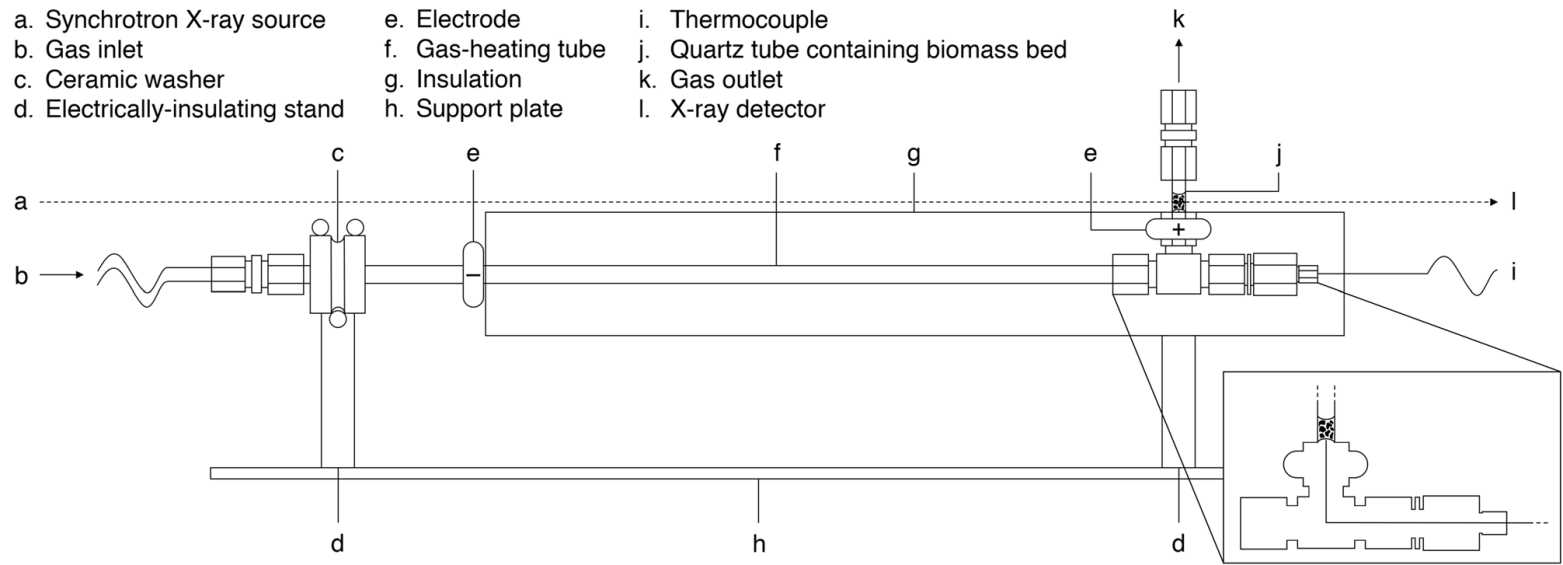
Schematic of the novel purpose-built reactor used to acquire in-situ radiographs.

**Figure 9:**
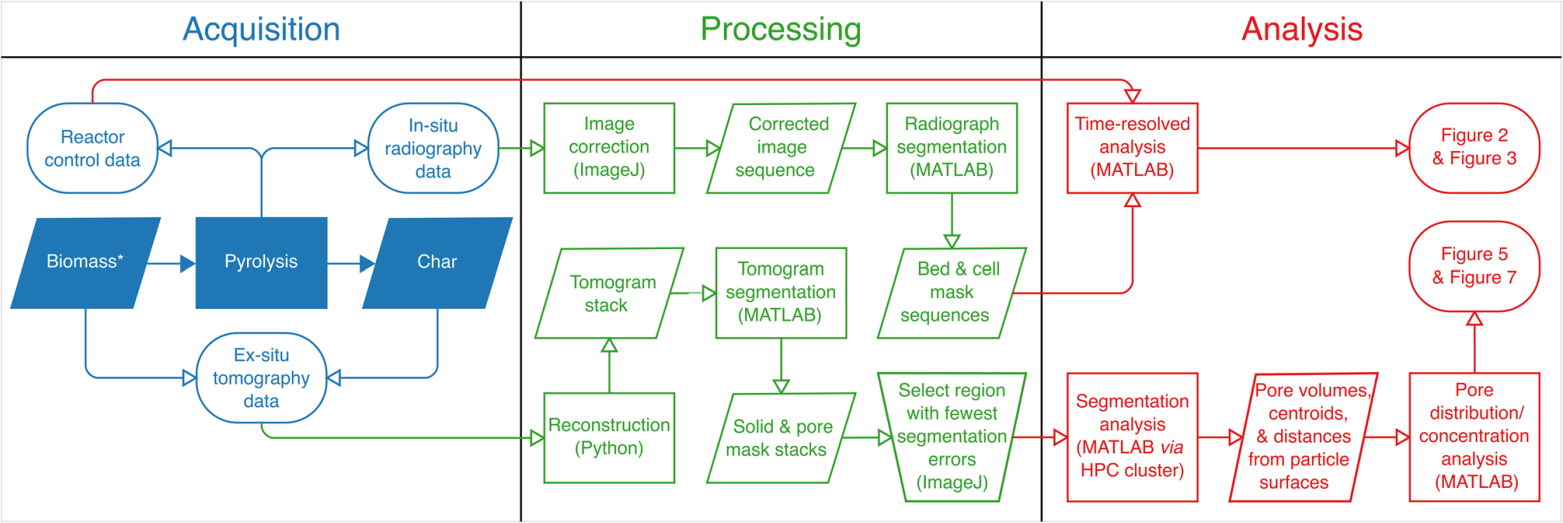
Pipeline diagram for data acquired during and after pyrolysis. Symbol meanings follow ISO 5807:1985: Information Processing^59^. Filled symbols represent physical systems; unfilled symbols represent data systems. MATLAB source code is available at: 10.5281/zenodo.3568050. *Raw biomass was not imaged before pyrolysis, but rather representative raw samples were imaged along with chars after pyrolysis. HPC: High-Performance Computing; here “remote HPC cluster” refers to Queen Mary's Apocrita HPC facility.

Experiments were performed at the Diamond-Manchester Imaging Branchline I13-2^53,54^ of Diamond Light Source (Oxfordshire, UK). A partially-coherent, near-parallel, polychromatic ‘pink’ beam (1.3 mm pyrolytic graphite & 3.2 mm aluminium filters; 8-30 keV; weighted mean ~ 27 keV) was used for both 2D imaging (radiography) and 3D imaging (tomography). Images were phase-contrast enhanced, with a propagation distance of ~ 40 mm, and collected by a detector (pco.edge 5.5—PCO AG, Germany; sCMOS sensor of 2560 × 2160 6.5 μm pixels) mounted atop a scintillator-coupled microscope of variable magnification. Magnification was set to give an effective pixel size of 1.625 μm for data collection, using a 500 μm LuAG:Ce scintillator. Beds of biomass (6 mm tall) were fixed between two stainless steel meshes in 3 mm inner diameter, 1.5 mm thick quartz tubes. Using a novel purpose-built pyrolysis reactor (See Fig. 8 for schematic), beds were convectively heated by a 3 L min^−1^ stream of resistively preheated argon at a rate of 6 °C min^−1^ to peak temperatures of 250, 350, and 450 °C. Beds were held at peak temperature for 30 min before cooling to 70 °C under the same gas flowrate. During this pyrolysis process, radiographs of the top 3.5 mm of the 3 mm wide cell were acquired in a single plane. After pyrolysis, tomographic data were acquired for chars and representative raw samples. Images were acquired at equally-spaced angles over 180° of continuous rotation (‘fly scan’), with an extra projection (not used for reconstructions) collected at 180° to check for possible sample deformation and bulk movements relative to the first (0°) projection^55^. Reconstructions were generated with the open source, modular pipeline Savu^55^. Images were first normalised via flat- and dark-field correction, followed by corrections for optical distortions^56^ and ring artefacts^57^. Prior to reconstruction via filtered back projection, a Paganin filter was employed with a δ/β ratio of 25. The pipeline used to process and analyse the acquired data is diagrammed in Fig. 9. Subsequent steps, including segmentation and analysis, relied entirely on custom MATLAB code, which has been made publicly available in full under an open-source license^58^.

### Statistics

Single sample beds for each condition were considered, and although each contained several particles, beds were analysed as a whole, so no significance testing between conditions could be performed. Here, instead, all statistical transformations applied to the data presented in this study are disclosed. Radiography data were smoothed using a moving average filter. Derivative radiography data were smoothed using a low-pass differentiation filter. Particle beds were cropped in the X–Y plane by the minimum amount necessary to account for any tilt in the imaging cell, and only 2 mm sections in the Z plane containing the fewest segmentation errors (as determined visually using ImageJ) were considered. Pores with volumes more than a standard deviation greater than the mean pore volume in each bed were excluded from pore volume distribution analyses because their centroids would not accurately reflect their locations. Finally, pore volume distributions were smoothed using a LOESS (locally estimated scatterplot smoothing) filter.

## Data Availability

The data that support the findings of this study are available from the corresponding author upon reasonable request.

## Code availability

The custom code used to process and analyse the data that support the findings of this study is available under a GNU Affero General Public License v3.0 via Zenodo with the identifier https://doi.org/10.5281/zenodo.3568050.
